# Patient-Centric Approach to Personalized Electronic Medical Records via QR Code in Japan

**DOI:** 10.2196/57332

**Published:** 2024-12-23

**Authors:** Yoshihiko Izumida, Takashi Omura, Masahiro Fujiwara, Shoko Nukaya, Akio Yoneyama, Sow Boubacar, Shinichiro Yabe, Rika Noguchi, Shima Nakayama, Wataru Muraoka, Yuki Okuno, Sho Miyashita, Yurika Ishihara, Yuto Moriwaki, Ryoji Otani, Junichiro Adachi, Kenichiro Tanabe, Yoshihisa Yamano, Yasushi Takai, Masaru Honjo

**Affiliations:** 1 Department of Endocrinology and Diabetes Saitama Medical Center Saitama Medical University Kawagoe, Saitama Japan; 2 Life Science Laboratories KDDI research atelier KDDI Research, Inc Fujimino, Saitama Japan; 3 Research DX Center Tohoku Forum for Creativity Tohoku University Sendai, Miyagi Japan; 4 Center for Maternal-Fetal and Neonatal Medicine Saitama Medical Center Saitama Medical University Kawagoe, Saitama Japan; 5 Department of Pathophysiology and Bioregulation St.Marianna University Graduate School of Medicine Yokohama, Kanagawa Japan; 6 Department of Neurology St.Marianna University School of Medicine Yokohama, Kanagawa Japan; 7 Department of Obstetrics and Gynecology Saitama Medical Center Saitama Medical University Kawagoe, Saitama Japan

**Keywords:** Sync for Science-J, S4S-J, electronic medical record, personal health record, privacy preference manager, patient-generated health data, Health Level 7 Fast Health Care Interoperability Resources, HL7-FHIR, logical observation identifiers names and codes, LOINC, open science, mobile health, app, digital health, digital intervention

## Abstract

Government policies in the United States and the European Union promote standardization and value creation in the use of FAIR (findability, accessibility, interoperability, and reusability) data, which can enhance trust in digital health systems and is crucial for their success. Trust is built through elements such as FAIR data access, interoperability, and improved communication, which are essential for fostering innovation in digital health technologies. This Viewpoint aims to report on exploratory research demonstrating the feasibility of testing a patient-centric data flow model facilitating semantic interoperability on precision medical information. In this global trend, the interoperable interface called Sync for Science-J (S4S-J) for linking electronic medical records (EMRs) and personal health records was launched as part of the Basic Policy for Economic and Fiscal Management and Reform in Japan. S4S-J controls data distribution consisting of EMR and patient-generated health data and converts this information into QR codes that can be scanned by mobile apps. This system facilitates data sharing based on personal information beliefs and unlocks siloed Internet of Things systems with a privacy preference manager. In line with Japanese information handling practices, the development of a mobile cloud network will lower barriers to entry and enable accelerated data sharing. To ensure cross-compatibility and compliance with future international data standardization, S4S-J conforms to the Health Level 7 Fast Health Care Interoperability Resources standard and uses the international standardized logical observation identifiers names and codes (LOINC) to redefine medical terms used in different terminology standards in different medical fields. It is developed as an applied standard in medical information intended for industry, health care services, and research through secondary use of data. A multicenter collaborative study was initiated to investigate the effectiveness of this system; this was a registered, multicenter, randomized controlled clinical trial, the EMBRACE study of the mobile health app M♡Link for hyperglycemic disorders in pregnancy, which implements an EMR–personal health record interoperable interface via S4S-J. Nevertheless, the aforementioned new challenges, the pivotal Health Level 7 Fast Health Care Interoperability Resources system, and LOINC data mapping were successfully implemented. Moreover, the preliminary input of EMR-integrated patient-generated health data was successfully shared between authorized medical facilities and health care providers in accordance with the patients’ preferences. The patient-centric data flow of the S4S-J in Japan is expected to guarantee the right to data portability, which promotes the maximum benefit of use by patients themselves, which in turn contributes to the promotion of open science.

## Introduction

### Global Convergence in Digital Health Infrastructure

The standardized medical information network in Europe and the United States not only provides high-quality medical care in individual practice but also serves as a development research infrastructure that enables effective epidemiological research. When digital health care is provided with appropriate infrastructure, training, and engagement, it has the potential to improve population health by using artificial intelligence, big data, and precision medicine [1]. FAIR (findability, accessibility, interoperability, and reusability) data practices can enhance trust in digital health systems, which is crucial for their success. Trust is built through elements such as FAIR data access, interoperability, and improved communication, which are essential for fostering innovation in digital health technologies [2]. The ELIXIR Hub of the European Committee (EC) integrates Beacon to connect geographically distributed data centers and unify their data access methodologies [3,4]. The cloud-based analysis platform enables ready sharing and reproduction of research results through workspaces in the National Institutes of Health All of Us research program’s Researcher Workbench [5,6]. With continued adoption, these systems are creating a large network of globally searchable data sets that have the potential to unlock new data-driven discoveries and applications in medicine. Meanwhile, in the policy making of medical information, the European Commission initiated policies on cross-country data sharing of individual-level data, specifically through initiatives like the European Health Data Space in March 2022 [7], and the Data Governance Act, a new EC legal system, came into effect in September 2023, promoting standardization by the EC, as well as the creation of value in the use of FAIR data in anticipation of improved computational technology [8]. The legal system is being developed to promote data-driven innovation, as well as to develop low-cost medical technology. The US Department of Health and Human Services, through the Office of the National Coordinator for Health IT, released the draft 2024-2030 Federal Health IT Strategic Plan which places an emphasis on addressing the policy and technology components essential for securely catering to the diverse data requirements of all health IT users [9]. In response to this global trend, the Basic Policies for Economic and Fiscal Management and Reform 2022, approved by the Japan cabinet office on June 7, 2022, set forth a policy of linking health and medical data obtained within and outside medical institutions. Consequently, the Japan cabinet office devised a national medical information platform to encompass a broad range of data, including medical and long-term care information, medical receipts, data on specific medical examinations, vaccination information, electronic prescription data, and data on municipality clinical examinations, as well as electronic medical records (EMRs). The objective of this initiative is to facilitate the adoption of online medical care and to promote the implementation of artificial intelligence hospitals with a view to advancing medical digital transformation. Furthermore, the platform will examine standard EMRs and use EMR data in a manner that optimizes treatments, facilitates the development of new artificial intelligence–based or other medical technologies, and supports drug discovery [10].

### Economic and Clinical Impact of Mobile Health Technologies

In line with this policy, this research group was commissioned by the Japan Agency for Medical Research and Development to develop a mobile health (mHealth) app M♡Link. It designed and implemented the Sync for Science-J (S4S-J) interface to realize EMR-personal health record (PHR) interoperability using a QR code. In this research project, we implemented this system to construct a mobile cloud network designed for Japan’s medical situation that enables information sharing centered on individuals and enables data sharing based on personal free choice with a privacy preference manager (PPM) [11] for siloed Internet of Things (IoT) systems. Putting everything in perspective to cover all steps in a FAIR data management plan when conducting health research efforts, 56.57% of the time could be saved when using the FAIR4Health solution. This translated into economic savings of €16,800 (€1=US $1.0821 in 2023) per month, representing 17.11% of the cost per person per month of the institution [12].

Recently, the widespread use of internet technologies and mHealth tools for public health and medical purposes has changed human life [13,14]. SMS text messages sent from cell phones using reminder systems had a positive effect on medication adherence among patients with chronic diseases and those in need of medical services [15,16]. mHealth apps have been widely applied in the medical management of patients with cancer [17], diabetes [18,19], cardiovascular disease [20,21], and other chronic conditions [22]. Obstetric and gynecologic practices are also increasingly equipped with telemedicine to improve patient access to health care services. Previous studies have demonstrated the effectiveness of telemedicine services in obstetrics and gynecology, including improved health behaviors [23,24], weight management [25], mental health problems [26], and postpartum depression [27] in pregnant women. The effects of telemedicine as another applied medical infrastructure have also been reported to be associated with improved health outcomes for high-risk obstetric patients, including hypertensive disorders, diabetes, fetal abnormalities, and pregnancy in underserved areas [28].

### Evolution of FAIR-Compliant Interoperability Standards Through HL7-FHIR and SMART Technologies

The critical challenge of health care data interoperability, a key issue in modern health care systems, the increasing volume of health data necessitates efficient data-driven approaches to improve various aspects of health care, including disease detection, prognosis, clinical research, and patient management. However, the current landscape is characterized by data silos and incompatible systems, hindering the seamless exchange of information. Amid this current of events, the components of FAIR digital objects can be represented in Health Level 7 Fast Health Care Interoperability Resources (HL7-FHIR), thus increasing their interoperability and reusability. Appropriate FHIR profiles can further facilitate advanced search capabilities based on extensive metadata, and easier access to the health data capitalizing on the data model of HL7-FHIR [29]. Consequently, interoperability develops a communication framework between noncommunicable systems, which can be achieved by transforming health care data into ontologies. Thus, an effective solution is the development of methods for finding matches among the various components of ontologies in health care, in order to facilitate semantic interoperability [30]. The SMART (Substitutable Medical Applications and Reusable Technologies) team initially selected platform components that suited our mission, emphasizing web standards (eg, HTML, JavaScript, OAuth, and resource description framework) and widely adopted terminologies for coded data (eg, RxNorm [31], logical observation identifiers names and codes [LOINC] [32], and Systematized Nomenclature of Medicine—Clinical Terms) [33]. The remaining need was a standard for sharing granular clinical data (eg, a prescription, lab result, or blood pressure measurement) with a developer-friendly, semantically consistent, on-demand (vs server-initiated messaging) approach. To expose patient-level data according to these models [34], they built a corresponding representational state transfer application programming interface (API) offering data access through a fixed URL structure and HTTP verbs (eg, GET and POST) [35]. We added and refined our set of clinical statements and API calls over time to enable richer payloads with more expressive queries, which was achieved by transforming health care data into ontologies. An effective solution to this problem is the development of methods for finding matches among the various components of ontologies in health care, in order to facilitate semantic interoperability [36]. The patient-centric approach through the S4S-J in Japan is expected to guarantee the right to data portability, which is a patient right, and at the same time promote the maximum benefit of the utilization by the patients themselves. Therefore, we have started clinical studies of the mHealth app M♡Link for hyperglycemic disorders in pregnancy, which implements this EMR-PHR interoperable interface S4S-J on the data model of HL7-FHIR. This viewpoint aims to report on exploratory research demonstrating the feasibility of testing a patient-centric data flow model facilitating semantic interoperability on precision medical information.

## Architecting Japan’s Digital Health Evolution

### Stratified Health Data in Japan

**Figure 1:**
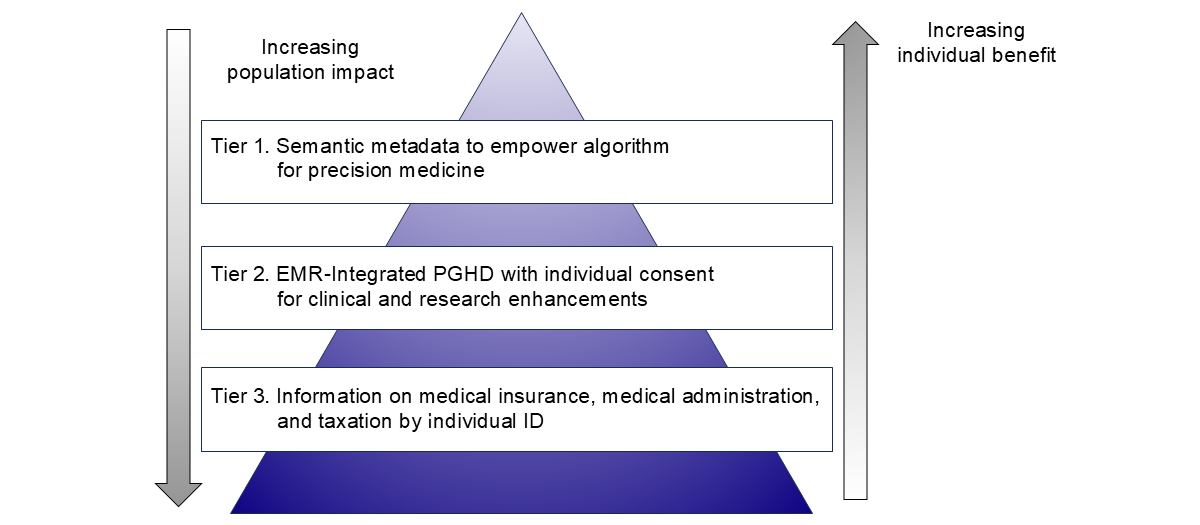
Stratified health data in Japan. Decreasing tiers represent information that has a stronger impact on the public, whereas increasing tiers represent information that offers higher individual benefit. Further digitization is currently underway with the objective of semantics, and efforts are being made to address issues of data collection and traceability in a way that is easily understandable. DPC: diagnosis procedure combination; EMR: electronic medical record; HL7-FHIR: Health Level 7 Fast Health care Interoperability Resources; ICD: International Classification of Disease; LOINC: Logical Observation Identifiers Names and Codes; NDB: national database; PGHD: patient-generated health data; PRO: patient-reported outcome; SNOMED-CT: Systematized Nomenclature of Medicine-Clinical Terms.

In the health care industry, large standards-developing organizations have defined numerous standards, data formats, terminologies, etc, in order to support the design and building of interoperable IT systems. Perhaps the most well-known and most important standards are the ones introduced by HL7, which can be used as a foundation for the development of data exchange standards among eHealth systems [37]. In 2022, the Ministry of Health, Labor, and Welfare established HL7-FHIR as a medical data standard regulation with the objective of promoting the interoperability of medical data and implementing the use of FAIR data [38]. In accordance with this Japanese government policy, we developed an API with the objective of achieving interoperability between EMR and PHR data. This was based on the HL7-FHIR standard, with the use of the LOINC terminology. This interface is distinguished by two key features: a patient-centric data flow enabled by dynamic e-consent named PPM, which facilitates personal data use permissions, and the use of QR codes, which are specifications readily implementable in the Japanese information environment for data transport. Moreover, metadata uses LOINC, a terminology focused on laboratory observations [36], in response to the functional requirements of M♡Link-app embedded S4S-J. Additionally, a data library has been implemented, converted from the Japan Laboratory Analysis Code standard to LOINC, which enhances the functionality of semantic interoperability. Consequently, in this study, we have developed an M♡Link using S4S-J and verified the effectiveness of the system. S4S-J is an interface for linking the warehouse data consisting of EMR and patient-generated health data (PGHD) and converting it into a QR code that can be scanned into a mobile app. S4S-J complies with the HL7-FHIR standard and unifies medical language with globally standardized terminology using LOINC. Developed as an applied standard in the medical information business, S4S-J is intended for medical applications in cooperation with Mynaportal, Japan’s Public Individual Number Card Service, and development research and health care services leveraging clinical information through secondary use of data. Japanese medical information is stratified as follows (Figure 1 and Textbox 1). Tier 3 provides public information on taxation, public medical insurance, and public health checkup systems through the Individual Number Card Service; Tier 2 provides FAIR data based on EMR-integrated PGHD with individual consent for the enhancement of research; and Tier 1 provides semantic metadata empowers algorithm for precision medicine. In the context of the rich metadata objects proposed by Haslhofer and Klas [39], it has been suggested that metadata instances may be extended using annotated semantic descriptors drawn from various sources, including different ontologies, terms, and coding systems. Meanwhile, in this study, the priority was on implementation and an approach was selected based on an outline and classification of metadata interoperability technology that would be optimal for the specific metadata integration scenario. S4S-J controls data distribution mainly for Tier 1 through Tier 2, making it facilitating the provision of purpose-built medical services to users, and at the same time promoting the industrial secondary use of data for drug discovery research. S4S-J and structured metadata will be used to provide precise telemedicine to pregnant women with hyperglycemic disorders in pregnancy using the mHealth app M♡Link, and a clinical registry study will be conducted to evaluate the usefulness of the system.

**Three tiers of stratified health data in Japan.**

**Tier 1**

Semantic technologies provide a layer of metadata to content that allows computers to understand and make connections to it. The implementation of semantic metadata facilitates the enhancement of algorithms used for the computation of medical solutions, thereby advancing the field of precision medicine. In research and some commercial applications, the following data elements are being used to expand the metadata vocabulary with natural definitions: (1) standardized medical terms and ontologies such as Systematized Nomenclature of Medicine—Clinical Term, International Classification of Diseases, and Logical Observation Identifiers Names and Codes; (2) omics analysis, including genome, transcriptome, proteome, metabolome, and metagenomics; (3) amino acid composition, fatty acid composition, vitamins, trace metals, etc, as dietary nutrients; and (4) external environmental factors.

**Tier 2**

Electronic medical record (EMR)–integrated patient-generated health data (PGHD) is in accordance with the Health Level 7 Fast Health care Interoperability Resources standard, and upon obtaining electronic consent for the use of personal information, it is accessible for utilization of primary data for telemedicine and mHealth. Furthermore, it will serve as a valuable source of secondary data for the development of new health care services and research initiatives in academic and industrial settings. The data set includes medical data, such as patient interviews, blood biochemistry, and images (x-rays, computed tomography, magnetic resonance imaging, endoscopes, and ultrasound sonography), as well as PGHD (biological sensor data such as number of steps, sleep, oxygen saturation, blood pressure, body temperature and electrocardiogram, nutritional intake data, patient-reported outcomes (PROs), and electrograms). Additionally, the data set incorporates nutritional intake and PROs.

**Tier 3**

Information on medical insurance, health administration, and taxation, based on Mynaportal, the government’s individual number in Japan. The Mynaportal is only active for specified purposes as defined by legislation: (1) social security, including pensions, medical insurance, and welfare services; (2) taxation, including income tax returns and withholding certificates; and (3) disaster policy, including support for disaster survivors. The preliminary set of medical data serves as a reference point for the standardization of data exchange between EMRs. It encompasses the medical information provision form, discharge summary, health checkup result report, disease or injury name, allergy, infectious disease, contraindicated drugs, chemical examinations, and prescription information. The data comprising Tier 3 is stored in the NDB, and the statistical data are accessible to the public. These include (1) diagnosis procedure combination data, (2) information on specific health checkups, and (3) health guidance.

### An EMR-PHR Medical Information Interoperable Interface: S4S-J

**Figure 2:**
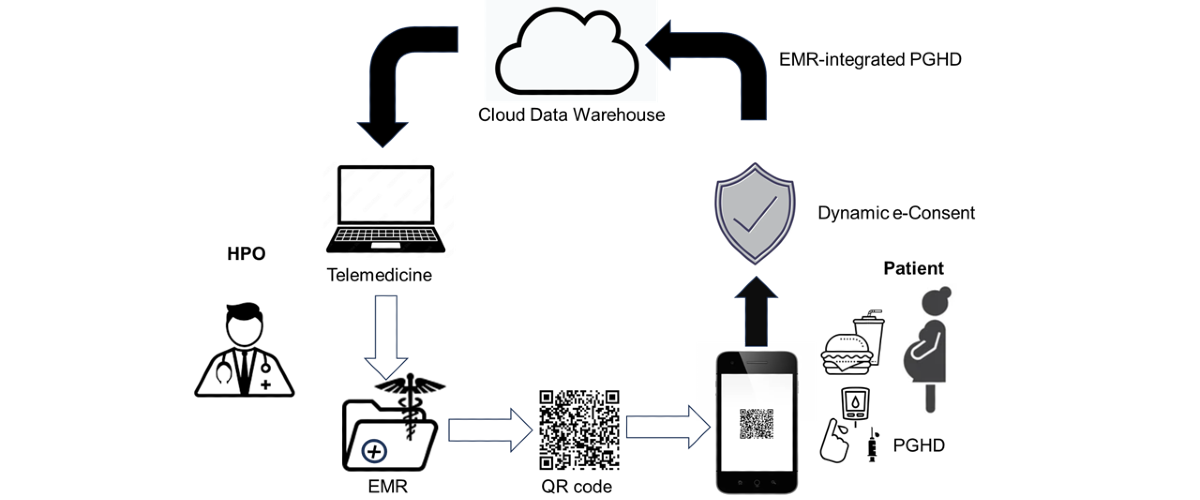
The information circulation on S4S-J interface EMR standardized in the HL7-FHIR, SMART health cards and oneM2M aligned LOINC terminology is converted to a QR codes. The QR coded EMR is captured by the patient's mobile app and processed with PGHD into a JSON bundle according to the Smart health cards standard to output structured EMR-integrated PGHD, which is stored in a cloud data warehouse. Healthcare providers download the metadata and EMR-integrated PGHD via the cloud data warehouse and access highly detailed and rich clinical data to provide precise telemedicine. In the aspect of research promotion, data distribution with the consent of personal information use is available through S4S-J, and it is easy to analyze aggregated EMR-integrated PGHD as a resource for research and development.

**Figure 3:**
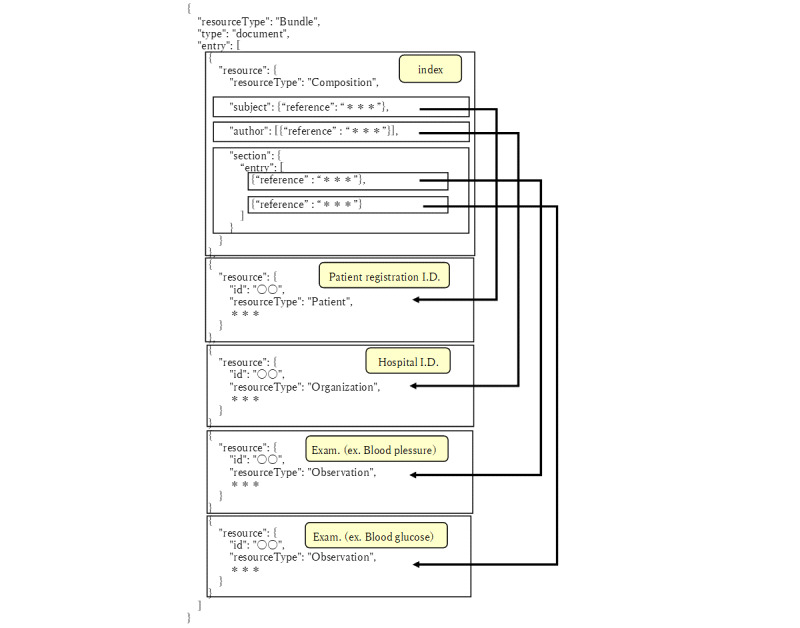
The FHIR bundle consists of EMR-integrated PGHD. EMR-integrated PGHD are converted into JSON bundles in compliance with the HL7-FHIR and SMART health cards standards. The medical information contained in the payload portion of the Smart Health Cards, such as “hospital,” “patient,” and “infectious disease test,” are described as an array in the “entry” field of the “bundle” resource in HL7-FHIR. The entire data is bundled as a “bundle” resource, and the resource type is “document” according to the Medical Information Form HL7FHIR Description Specification (Version 1). The “composition” resource at the beginning of the “entry” in the “bundle” serves as the table of contents contained in the bundle. Behind it, medical information expressed as resources are lined up. Medical and laboratory data such as “blood pressure” and “blood sugar” are represented as “observation” resources. EMR: electronic medical record; HL7-FHIR: Health Level 7 Fast Health care Interoperability Resources; PGHD: patient-generated health data; SMART: Substitutable Medical Applications and Reusable Technologies.

**Figure 4:**
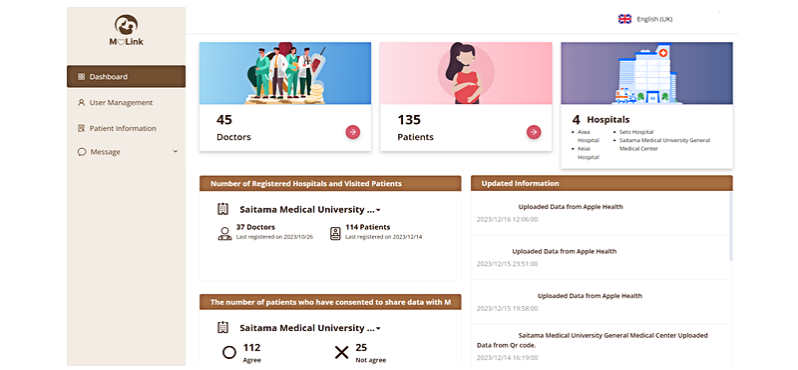
UI/UX of the mHealth web app system M♡Link. User interface for data sharing indicating the number of patient consent forms obtained and HPs authorized to share their data. HP: health care provider; UI: user interface; UX: user experience.

**Figure 5:**
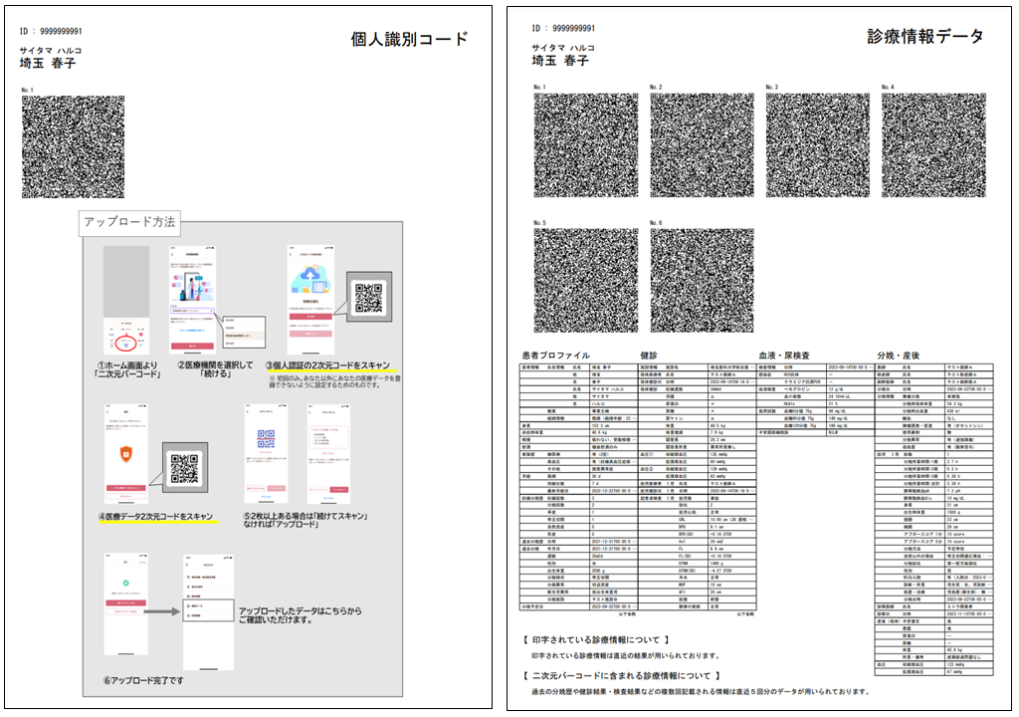
Upload of EMR-integrated PGHD to the cloud data warehouse via QR codes and the M♡Link app. The process is as follows: EMR-integrated PGHD are captured in the app through QR codes, displayed in the user interface or user experience, and stored in the cloud data warehouse. Encryption key to identify the individuals (left) and QR coded EMR-integrated PGHD (right). EMR: electronic medical record; PGHD: patient-generated health data.

**Figure 6:**
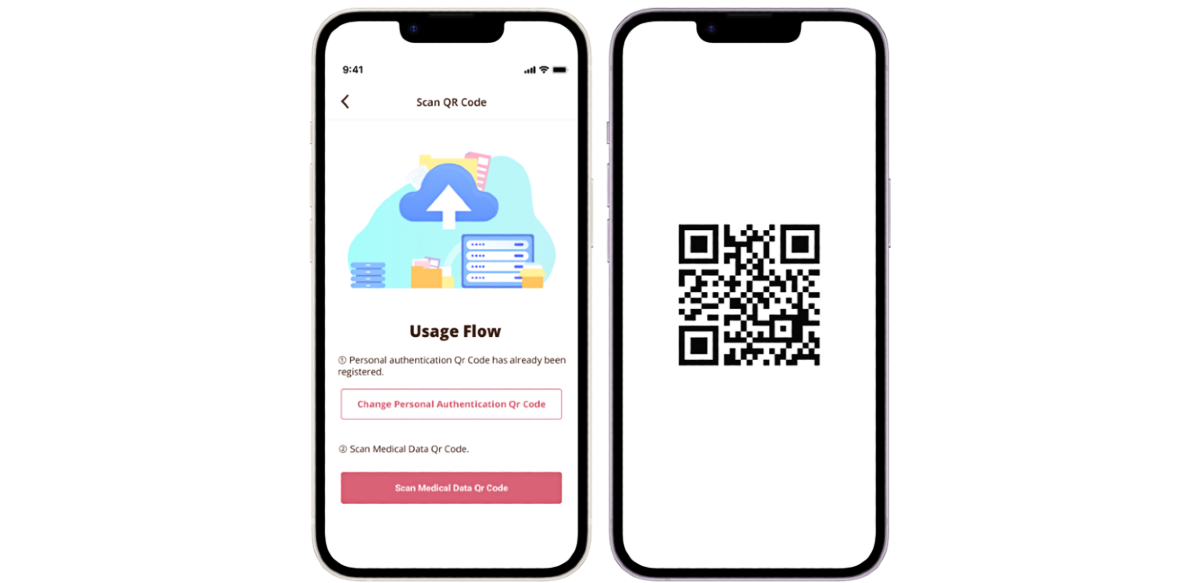
Uploading the EMR-integrated PGHD to the cloud data warehouse via QR codes. EMR-integrated PGHD are captured in the app through QR codes, displayed in the UI/UX, and stored in the cloud data warehouse. EMR: electronic medical record; PGHD: patient-generated health data; UI: user interface; UX: user experience.

**Figure 7:**
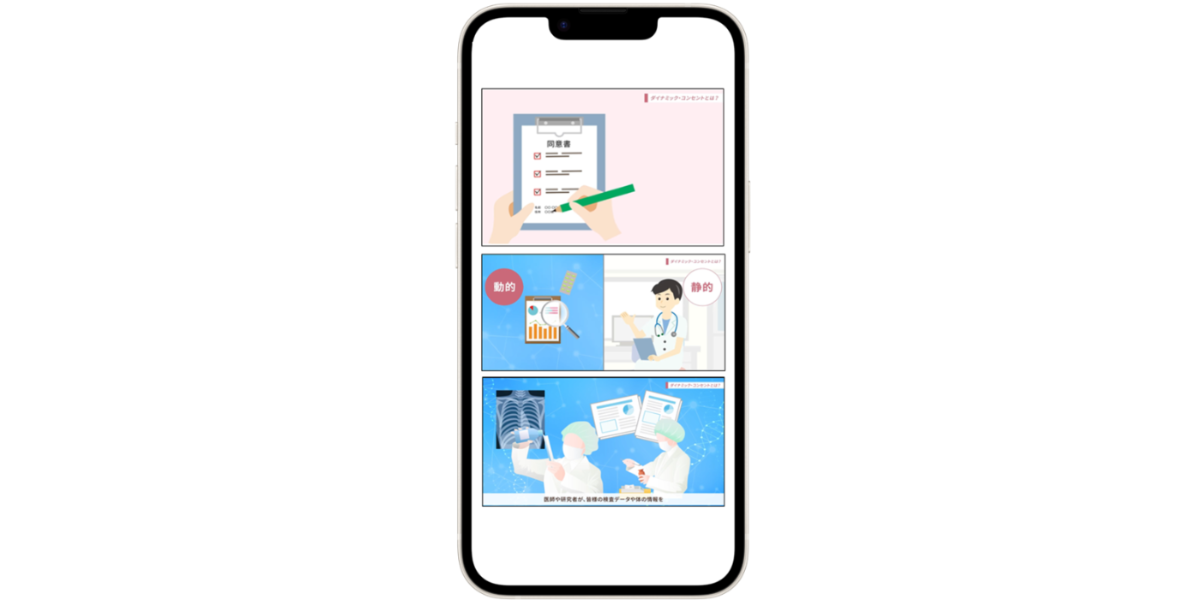
Displaying acquired dynamic e-consent. Explanatory movie for patients embedded in the mobile app explains how the data will be used, what users can access the wider medical service, and how data sharing will contribute to research and drug discovery through dynamic e-consent.

S4S-J is an interface that has three main functions tailored to the actual situation of medical information sharing in Japan. The system information structure and design of S4S-J are shown in Figure 2. The simple QR code system was specifically designed to interoperate the EMR among major obstetric medical record systems with the S4S-J cloud data warehouse. It covers the requirements of third-party organizations for the primary use of medical information by medical providers and medical services (eg, online medical care and interhospital sharing of medical information) and secondary use in research and development (eg, drug discovery research and development of new diagnostic equipment): (1) medical information sharing within QR-coded EMR, (2) conversion to LOINC as a global standard terminology, and (3) privacy data provision based on the consent of using personal information by PPM [11]. S4S-J exports the EMR of various EMR vendors in the form of QR codes by converting them into JSON bundles in compliance with the HL7-FHIR and SMART health cards standards. SMART health cards are verified versions of your clinical information, such as vaccination history or test results. They allow you to keep a copy of your important health records on hand and easily share this information with others if you choose. SMART health cards contain a secure QR code and may be saved digitally or printed on paper, which is encoded as compact serialization JSON web signatures (Figure 3) [40]. For enhanced security of personal information and to prevent accidental exposure, S4S-J implements SMART health cards compliant with RFC7515 (JSON web signatures) in a format enhanced with encryption based on RFC7516 (JWE) to prevent QR codes from being read by anyone other than the person in the QR code. The QR code, which is the key for personal identification, is output at the same time and can be scanned via mobile app to read the EMR when the personal code is authenticated and the verification is confirmed. (Figures 4 and 5). This eliminates unintended and dishonest access. Japan Laboratory Analysis Code, a clinical laboratory code established by the Japanese Society for Laboratory Medicine, and medical information and others are converted to the international standard terminology LOINC. The user can choose to share the data with health care providers, government health agencies, private services, and research and development organizations according to the preferences of the patient’s information use and contact various services. In this process, we have designed an easy-to-use interface to obtain informed consent electronically, using videos to provide clear explanations and aid in understanding. Consent can be withheld completely or temporarily for privacy with the ability for dynamic consent to adjust to changes in patient intent over time (Figures 6 and 7).

### A System Design and Information Framework for mHealth Solutions: The M♡Link App

**Figure 8:**
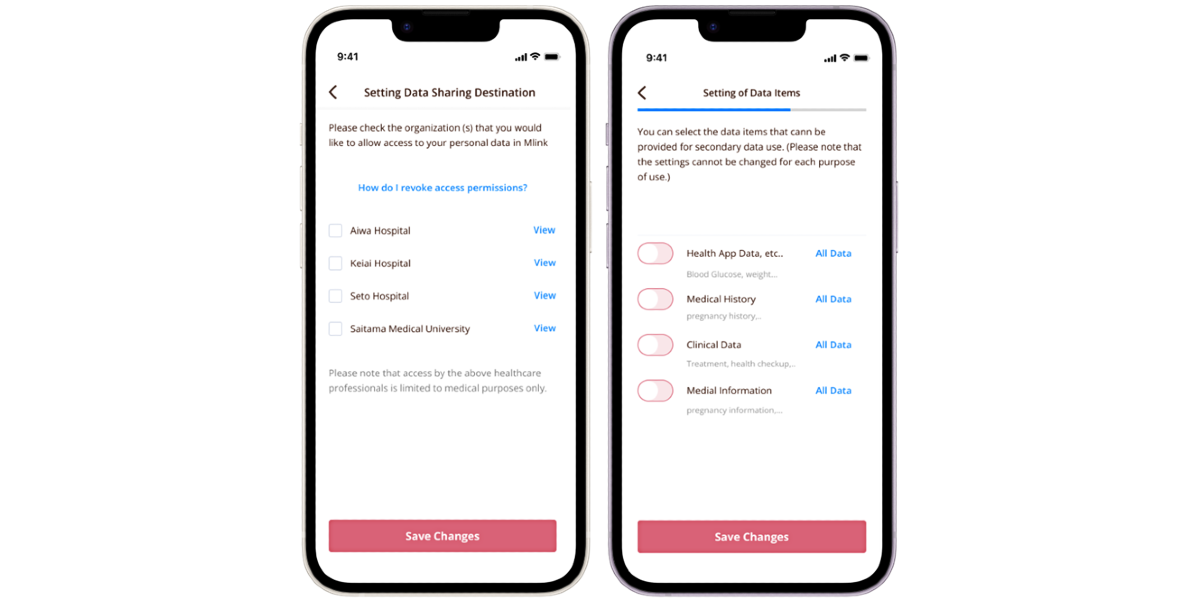
Setting up data sharing based on individual decisions through dynamic e-consent. Left: checkboxes allow selecting HPs with which to share data. Right: dynamic e-consent for secondary use of data; the UI/UX facilitates the secondary use of data by showing the attributes to be used, including PGHD, medical history, clinical biochemical values, ultrasound values, and clinical physical values, and the attributes of the recipients, such as HPOs, government health agencies, academia, industry, and other organizations. HP: health care provider; HPO: health care provider organization; PGHD: patient-generated health data; UI: user interface; UX: user experience.

The system was developed in a robust working environment with a well-detailed specification. During the development, we have used various tools and software to ensure security and efficiency in the system. The development environment consisted of a cloud data warehouse built on Amazon Web Services. Other software tools such as Firebase (Google) for push notification services and Twilio [41] for messaging have been used to enhance the system’s capabilities. The hosting repository being used is GitHub, and to enable automated testing and deployment continuous integration and continuous deployment pipelines were implemented using Kubernetes (Google). The backend of the system was built using Hasura [42] which provides a ready-to-use GraphQL API on top of the PostgreSQL database [43]. PostgreSQL served as the primary database, chosen for its reliability and powerful querying capabilities. The web application frontend was developed using React.js and the ant-design library (Figure 8). We also have developed the Android and iOS versions using React Native, a well-known JavaScript library for building hybrid mobile apps. The system integrated various third-party APIs and services to enhance its functionality. This included the use of “Calomeal API” [44] which is a food image analysis and which incorporated an API. The system also used the React Native Healthkit package to enable synchronization with Google Fit and Apple Health. We have been performing regular security audits including cloud application security assessments and network vulnerability assessments to identify potential threats. In terms of use, the system collects various types of PGHD, such as blood glucose levels, insulin dosage, and nutrient and calorie intake. It also includes medical records like ultrasound fetal predicted weight and maternal weight, and the medical information is mapped using HL7-FHIR format and converted into QR codes. This allows for easy capturing and incorporation of data into the cloud via the mobile app. Users can scan QR codes to register their medical information. With user permission, this information can be shared with health care providers through the web application. All data, including symptoms, nutrient intake, and medication-related behaviors, are stored in a cloud data warehouse.

## Patient-Centric Digital Health Implementation in Maternal Care

### End-to-End Quality Evaluation for Patient-Centric Data Flow in the S4S-J and M♡Link App Architectures

An end-to-end evaluation was conducted from the user’s perspective to ascertain the quality control of each system component as an integrated ecosystem demonstrating the functionality of the patient-centric data flow. In particular, the patient-centric data flow was implemented in accordance with the following customer journey. First, health care providers enter the patient’s medical data into the EMR and upload data such as ultrasound measurement findings and biochemical test results. Medical information from EMRs is converted into QR codes compliant with S4S-J and generated in the EMR systems of four major EMR vendors for obstetrics and gynecology in Japan (MITLA Co, Ltd, NEWWAVE Co Ltd, TAK Co, Ltd, and TOITU Co, Ltd) at multiple medical facilities. Second, the QR codes were scanned on the M♡Link app, and the PGHD, comprising nutritional intake, blood glucose levels, insulin administration information, and patient-reported outcomes, were entered. Third, at multiple medical institutions, health care providers who had obtained consent for the primary use of patient data entered this data into the M♡Link web app screen and proceeded to provide medical care based on the large volumes of precise data.

In consequence, the end-to-end evaluation revealed issues that had not been identified during the system test. These issues are: (1) the attributes for standardized data in the data libraries used by each EMR vendor differed, resulting in discrepancies between companies and varying definitions of the details of medical information in some cases. (2) The presence of multiple QR codes within the same field of view when scanning a single QR code resulted in the interference of the recognition process, whereby the individual codes were not recognized as a single piece of information. Accordingly, the configuration of the QR code required refinement, and the precision of the reading functionality also necessitated enhancement. (3) In some instances, the reliability of certain entries, specifically “measurement time” and “before or after meal,” in the PGHD was not optimal. This led to the modification of the user interface and user experience to a function that automatically determined the label based on the time. The patient-centered data flow was confirmed to be functioning while maintaining consistency with the functions of the S4S-J API, following the overcoming of the obstacles. Moreover, it was demonstrated that this API optimizes the functions of the M♡Link app and aligns with the functional requirements of various obstetrics and gynecology EMR vendors, facilitating an effective and streamlined information flow.

### Implementation of the Pilot Study

Since 2022, a multicenter collaborative registry study (jRCT1030220452) and since 2023, a multicenter randomized controlled trial, the Efficacy of M♡Link based telemedicine on GDM in a randomized controlled trial (EMBRACE jRCT1032230258), have been conducted after receiving research ethics approval of Saitama Medical University, Saitama Medical Center, Clinical Research Review Committee (SOU2023-014). A mobile cloud network was established between three obstetrics and gynecology hospitals and a core university hospital to facilitate data collection and analysis. The participants are women with hyperglycemic disorders in pregnancy, and we have established a treatment style in which the pregnant women receive concurrent medical outpatient treatment via web-based medical service while attending obstetrics and gynecology departments. Using this app, online medical care in the form of mHealth, real-time blood glucose levels, nutrient and calorie intake, and medical information from the EMR-derived OB/GYN, such as estimated fetal and maternal weight, are displayed for diagnosis and decision-making by health care providers, as well as medical instructions for insulin dose adjustment and nutrient intake. Text-style messages were exposed via the app 1-2 times per week, sometimes as needed. In order to facilitate in-depth counseling, health care providers have implemented the use of the app through SMS text messages, with the objective of achieving high app retention rates. These strategies are designed to enhance pregnant women’s motivation for treatment and adherence to app use. The data disclosed and provided by patients as PGHD and the number of steps taken, as well as QR code-generated EMR and the hierarchical metadata could be generated.

The first feature is that the participants were pregnant women, a homogeneous target population, which tends to minimize variation in information and communication technology (ICT) literacy and makes the study design less susceptible to individual differences in app use skills. Even in the differences between genders, it has been shown in previous studies examining gender differences in ICT literacy that this is a significant difference in favor of women scoring higher on cognitive measures of computer use frequency, perceived ICT skills, and attitudes toward computers [45]. Compliance was also high, despite a limited understanding of gestational diabetes mellitus and dietary requirements and why changes were necessary [46]. Moreover, almost without exception, patients negatively recalled the process of measuring blood glucose multiple times a day and taking medications during pregnancy but recognized and showed resilience in making this work for the baby’s health. Women reported that health care providers could recognize that maternal health increases motivation for lifestyle changes during pregnancy that directly affect the baby [47]. As in previous reports, pregnant women actively participated in telemedicine using the app and then received mHealth on a patient-centric data flow infrastructure.

## Discussion, Issues, and Limitations

### Patient-Centric Approach to EMR-PHR Integration Within Social and Technical Constraints

In Japan, standardization of data distribution was adopted in 2022 as a standard by the Ministry of Health, Labor and Welfare, and is integrated in the HL7-FHIR standard, aiming to spread standardized electronic medical information and exchange systems. As a pilot operation, 3 documents and 6 pieces of information are provided: medical information including the name of injury or disease, allergy information, information on infectious diseases, and information on contraindications to drugs; examination information (tests useful in emergency situations, tests related to lifestyle-related diseases); and prescription information. For example, documentary information includes the following forms: medical information form, discharge summary with key images, and health examination results report. Nevertheless, the regulation of interoperable common API in Japan, defined as Sync for Science (S4S) in the United States, has yet to be officially released by the relevant bureau or enterprise organizations. Consequently, each tier of siloed data remains underused for research in academia and industry.

S4S-J is endorsed by the Japan Agency for Medical Research and Development and distributed by the Life Course Design Consortium, a multistakeholder consortium consisting of telecom, pharmaceutical companies, medical devices, academic organizations, and academia. This common interface that provides interoperability for EMR-PHR is compliant with HL7-FHIR and SMART health cards as standardization and enables the distribution of EMR and PGHD in a patient-centered manner. Because of avoiding subordinated to proprietary systems or applications defined by exclusive standards, S4S-J is characterized by converting EMRs to QR codes for patient-centered mobile circulation, which takes into account the tendency of notions that medical information stakeholders intend in Japan. Health care providers in Japan are hesitant to actively connect medical information to the IoT, preferring isolated networks due to concerns about cybersecurity and information disclosure [48]. Mobile and wearable devices are increasingly being developed for health care purposes. mHealth data includes personal health data collected from sensors and mobile apps. mHealth data and metadata standardization improves the ease of data aggregation and location accuracy (semantic interoperability) and reduces the cost of using this data for biomedical discovery, health improvement, and disease management [49]. Standardization of data along the entire PGHD integration pipeline is critical to ensure that it is device-independent, modular, flexible, versatile, and therefore, low-cost to integrate into clinical workflows [50]. For health care provider organizations, standardization will make it simpler to integrate data into workflows and write easily to EMRs for billing purposes. EMRs in the United States are currently required by law to support HL7-FHIR data and protocol standards [51]. As a schema to promote research and development. S4S has collaborated with Harvard Medical School, various EMR vendors, and the US Federal government (National Institutes of Health and the Office of the National Coordinator for Health IT). S4S is a technology that allows individuals to donate electronic clinical and health data to biomedical research studies, which provides the ability for participants to share their electronic health record–based clinical health data with selected research programs via an open, read-only API. The S4S API is based on the HL7-FHIR and OAuth 2.0 standards. The S4S API is being developed to allow applications to use OAuth 2.0 to provide read-only access to all or a portion of electronic personal health information about a patient available through the health care provider organization patient portal via the patient’s authentication credentials [52]. PGHD aggregation services that provide standards-based PGHD integration have a critical role to play in the transition from today’s siloed and friction-prone data ecosystem to a frictionless, interoperable system appropriate for patients. Health care leaders responsible for telemonitoring and other PGHD programs should explore and adopt a pipeline-based approach to standardize the integration of PGHD into clinical care [50]. Likewise, in Japan, data mapping with HL7-FHIR enables the integration of EMR-integrated PGHD, and data portability using QR codes provides the option of building a data flow that is adapted to Japanese usability. Moreover, the value sets are standardized using LOINC terminology. Meanwhile, since digital distribution in S4S-J is a patient-centric data flow, the amount of data distribution is limited by patient refusal to provide data and the frequency of data provision. The decline in adherence leads to loss of data provision opportunities and poor data quality. This means that health care providers need to encourage users to provide accurate data, record high-quality data in a text-based manner, and control the quality of data flow through frequent communication. Agreement and trust in data handling between patients and health care providers will enable the generation of well-structured, highly useful EMR-integrated PGHD and enable health care providers to provide precise health services based on a patient-centric data flow system. In that context, a key factor is for health care service providers to offer services that are attractive enough for patients to willingly provide their data, and to promote services that are mutually beneficial to both patients and health care providers in order to strengthen patient-centric data provision.

### Gender-Specific Advantages and Clinical Safety of Telemedicine Interventions

In considering application differences arising from gender differences in this study, the results indicated that young women outperformed men in the overall higher ICT literacy test score in a study that was administered to Norwegian students [53]. According to the results of multigroup confirmatory factor analysis, the test had sufficient measurement invariance across genders to allow the researchers to make meaningful comparisons. The comparison revealed that girls were more ICT literate than boys. The impact of gender differences in the use of ICT applications (annotated multimedia electronic readers) was also tested in pre- and postclassroom learning. The results show that women use learning tools more frequently than men [54]. These also suggest that the use of ICT applications for pregnant women may be a good indication and an effective treatment target.

As an intervention effect of the one-directional messages through a mobile phone, it significantly improved physical activity, healthy eating, and medication adherence in chronic heart disease patients. In addition, more people may also control cardiovascular disease risk factors by following the recommendations via messages. The intervention group was 62% more likely to control all five risk factors according to recommendations [55]. Likewise, in perinatal care, Text-4-Baby, another telemedicine service for pregnant women and its SMS text message service, was also associated with improvements in women’s health behaviors and attitudes toward parenting [56,57]. Thus, health outcomes in telemedicine could prove to be noninferior to traditional face-to-face care in the perinatal period. For example, the prenatal and newborn guidelines, education, and learning system of one of the largest telemedicine obstetric care programs providing telephone consultation services to pregnant women in the United States was as effective as traditional face-to-face services [58,59]. In the safety issue of telemedicine, programs involving synchronous videoconferencing were not associated with an increased risk of health complications or medical adherence compared to traditional outreach programs [56,60]. In a online obstetrics and gynecology consultation service in Japan, telemedicine services using multiple communication tools, such as chat messages and voice calls, have also been shown to be clinically safe [60]. The interim analysis of the registry using M♡Link suggests that it may improve postprandial blood glucose and improve target range (data not shown), however, there are limitations due to the small number of cases and the retrospective nature of the study and also suggests that further research is needed to evaluate the effect of telemedicine using EMR-integrated PGHD on the incidence of perinatal complications such as preterm birth or cesarean section.

### Limitations and Future Directions in Patient-Centric Health Data Systems

In S4S-J, metadata is mapped in accordance with HL7-FHIR standards, and instances are executed as part of the process. In this study, an objective evaluation was not conducted using statistical measures typically used to assess the reliability of agreement between multiple raters, such as Fleiss κ statistics [61]. This limits the ability to validate the appropriateness of metadata harmonization. The patient-centric data flow commences with the acquisition of consent to access personal data, which is contingent upon the appeal and consistency of the services provided as use cases. The study did not use statistical methods to verify the dropout rate or the reliability of the services provided, necessitating further analysis. In the implementation of data distribution adapted to usability in each country, it is important to introduce a system that ensures data security and reliability according to habitual backgrounds in digitalization. Over the future, a patient-centric approach will enable mHealth to effectively provide advanced health care services with EMR-integrated PGHD and metadata, both for the patients themselves and for health care providers and researchers.

### Conclusions

A QR code–based EMR-PGHD interoperable interface, S4S-J, was launched in the medical field in Japan. S4S-J constructs a mobile cloud network designed for Japan’s medical situation that enables data sharing based on personal choices with a PPM for siloed IoT systems. In accordance with Japanese information handling practices, the development of a mobile cloud network through S4S-J is expected to lower barriers to entry and accelerate information sharing. To ensure cross-compatibility and compliance with future international data standardization, S4S-J complies with the HL7-FHIR standard and unifies medical language using globally standardized terminology via LOINC. The patient-centric data flow of the S4S-J in Japan is expected to guarantee the right to data portability and promote a maximum benefit for patients themselves, which in turn will develop as an applied standard in health care services and contribute to open science through the secondary use of data in Japan. In the near future, a metadata integration scenario will be selected that allows for a generic metamodel approach to be used, with the objective of creating an information infrastructure that enables deep semantic understanding. In order to achieve this objective, it would be prudent to adopt a metamodel approach that is based on 5 key characteristics: extensibility, modularity, refinement, multilingualism, and machine processability [62]. Concurrently, efforts should be made to develop a sustainable information society through the implementation of green computing practices, in response to the growing concern over energy consumption and global warming [63].

## Abbreviations

API: application programming interface
EC: European Committee
EMR: electronic medical record
FAIR: findability, accessibility, interoperability, and reusability
HL7-FHIR: Health Level 7 Fast Health care Interoperability Resources
ICT: information and communication technology
IoT: Internet of Things
LOINC: logical observation identifiers names and codes
mHealth: mobile health
PGHD: patient-generated health data
PHR: personal health record
PPM: privacy preference manager
S4S: Sync for Science
SMART: Substitutable Medical Applications and Reusable Technologies
